# VMAT with DIBH in hypofractionated radiotherapy for left-sided breast cancer after breast-conserving surgery: results of a non-inferiority clinical study

**DOI:** 10.1093/jrr/rrad096

**Published:** 2023-12-12

**Authors:** Keiichi Jingu, Kengo Ito, Kiyokazu Sato, Rei Umezawa, Takaya Yamamoto, Noriyoshi Takahashi, Yu Suzuki, Keita Kishida, So Omata, Hinako Harada, Yasuhiro Seki, Nanae Chiba, Noriyuki Kadoya

**Affiliations:** Department of Radiation Oncology, Tohoku University Graduate School of Medicine, 1-1 Seiryo-chou, Aoba-ku, Sendai 980-8574, Japan; Department of Radiation Oncology, Tohoku University Graduate School of Medicine, 1-1 Seiryo-chou, Aoba-ku, Sendai 980-8574, Japan; Division of Radiation Technology, Tohoku University Hospital, 1-1 Seiryo-chou, Aoba-ku, Sendai 980-8574, Japan; Department of Radiation Oncology, Tohoku University Graduate School of Medicine, 1-1 Seiryo-chou, Aoba-ku, Sendai 980-8574, Japan; Department of Radiation Oncology, Tohoku University Graduate School of Medicine, 1-1 Seiryo-chou, Aoba-ku, Sendai 980-8574, Japan; Department of Radiation Oncology, Tohoku University Graduate School of Medicine, 1-1 Seiryo-chou, Aoba-ku, Sendai 980-8574, Japan; Department of Radiation Oncology, Tohoku University Graduate School of Medicine, 1-1 Seiryo-chou, Aoba-ku, Sendai 980-8574, Japan; Department of Radiation Oncology, Tohoku University Graduate School of Medicine, 1-1 Seiryo-chou, Aoba-ku, Sendai 980-8574, Japan; Department of Radiation Oncology, Tohoku University Graduate School of Medicine, 1-1 Seiryo-chou, Aoba-ku, Sendai 980-8574, Japan; Department of Radiation Oncology, Tohoku University Graduate School of Medicine, 1-1 Seiryo-chou, Aoba-ku, Sendai 980-8574, Japan; Department of Radiation Oncology, Tohoku University Graduate School of Medicine, 1-1 Seiryo-chou, Aoba-ku, Sendai 980-8574, Japan; Department of Radiation Oncology, Tohoku University Graduate School of Medicine, 1-1 Seiryo-chou, Aoba-ku, Sendai 980-8574, Japan; Department of Radiation Oncology, Tohoku University Graduate School of Medicine, 1-1 Seiryo-chou, Aoba-ku, Sendai 980-8574, Japan

**Keywords:** VMAT, DIBH, HF-WBI, postoperative radiotherapy, a feasibility study

## Abstract

The purpose of this study was to show the safety of volumetric modulated arc therapy (VMAT) with deep inspiration breath-hold (DIBH) in hypofractionated radiotherapy for left-sided breast cancer after breast-conserving surgery in a clinical setting. Twenty-five Japanese women, aged 20–59 years, who were enrolled in this prospective non-inferiority study received VMAT under the condition of DIBH with 42.4 Gy/16 fractions for whole-breast irradiation (WBI) ± boost irradiation for the tumor bed to show the non-inferiority of VMAT with DIBH to conventional fractionated WBI with free breathing. The primary endpoint was the rate of occurrence of radiation dermatitis of Grade 3 or higher or pneumonitis of Grade 2 or higher within 6 months after the start of radiotherapy. This study was registered with UMIN00004321. All of the enrolled patients completed the planned radiotherapy without interruption. The evaluation of adverse events showed that three patients (12.0%) had Grade 2 radiation dermatitis. There was no other Grade 2 adverse event and there was no patient with an adverse event of Grade 3 or higher. Those results confirmed our hypothesis that the experimental treatment method is non-inferior compared with our historical results. There was no patient with locoregional recurrence or metastases. In conclusion, VMAT under the condition of DIBH in hypofractionated radiotherapy for left-sided breast cancer after breast-conserving surgery can be performed safely in a clinical setting.

## INTRODUCTION

Postoperative whole-breast irradiation (WBI) following breast-conserving surgery has been a standard treatment strategy worldwide for early breast cancer [1]. For WBI after breast-conserving surgery, several randomized clinical trials showed that hypofractionated whole-breast irradiation (HF-WBI) achieved results comparable to those of conventional fractionated WBI [2, 3]. A Japanese clinical trial (JCOG0906) of HF-WBI showed that Grade 2 early adverse events occurred in 38 patients (12.4%) and no patients had Grade 3/4 events, and the results of that trial indicated that HF-WBI is safe for Japanese patients [4]. Previous studies showed increases in the incidences of acute myocardial infarction, ischemic heart disease, stenosis in the left anterior descending coronary artery, angina pectoris, pericarditis and valvular heart disease after left-sided radiotherapy compared with those after right-sided radiotherapy [5]. Deep inspiration breath-hold (DIBH) is a radiotherapy method in which patients take a deep breath before treatment and hold their breath while the radiation is delivered. In DIBH, the lungs fill with air and the heart moves away from the chest wall. Our planning study showed that DIBH and volumetric modulated arc therapy (VMAT) could minimize dosimetric parameters of the heart in patients with left breast cancer after breast-conserving surgery; however, VMAT slightly but significantly increased *V*_5_ (percentage of the organ receiving a dose of ≥5 Gy), *V*_10_ and *V*_20_ of the left lung [6]. The purpose of this study was to determine whether the combination of DIBH and VMAT can be safely performed in a clinical setting.

## MATERIALS AND METHODS

### Study design and endpoints

This trial was designed to evaluate the feasibility and safety of VMAT under the condition of DIBH in hypofractionated radiotherapy for patient with left-sided breast cancer after breast-conserving surgery.

### Patients

All of the patients had histologically proven breast cancer.

The patient selection criteria were (i) patients who received breast-conserving surgery for left breast cancer, (ii) patients who required postoperative radiotherapy, (iii) patients with Eastern Cooperative Oncology Group (ECOG) performance status of 0–1, (iv) 20–59 years of age, (v) patients who can hold breath for more than 10 s and (vi) patients who were willing to give written informed consent.

The exclusion criteria were (i) patients who required radiotherapy for a regional lymph node area, (ii) patients with other active cancers, (iii) patients with active collagenopathies, uncontrolled diabetes and serious cardiac, liver or pulmonary disease, (iv) patients with previous radiotherapy for the chest, (v) patients with synchronous bilateral breast cancer requiring adjuvant radiotherapy also for the right side, (vi) patients with active infections, (vii) patients who regularly use steroids, (viii) patients with a pacemaker or internal defibrillator and (ix) females who are pregnant, may become pregnant, have given birth within 28 days or are breastfeeding.

### Ethics

This clinical trial was approved by our institutional review board and ethics committee (approval number: 20531) and was registered at the University Hospital Medical Information Network Clinical Trials Registry (UMIN-CTR ID: UMIN 000043213).

### Radiation therapy

**Fig. 1:**
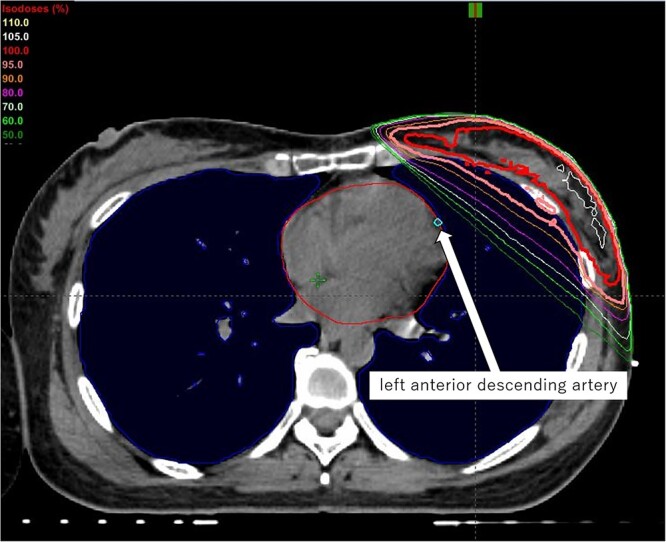
Example of dose distribution by VMAT with DIBH for the left breast.

All of the patients underwent DIBH CT scans at intervals of 2.5 mm in the supine position with their left arm above their head. All of the mammary glandular tissue and the tumor bed were delineated as the clinical target volume (CTV): the upper border was the second rib insertion, the lower border was at the plane where the glandular tissue disappears, the medial border was the lateral edge of the sternum and the lateral border was the lateral breast fold. The planning target volume (PTV) was generated by expanding the CTV with an additional 10-mm circular margin but within 2 mm of the skin surface. A VMAT plan used dual partial arcs of 40°–50° with a collimator angle of 30° and treatment couch angle of 0°, rotating in opposite directions. A dose of 42.4 Gy in 16 fractions covering 50% of the PTV was prescribed. The maximum dose rate was 1400 MU/min for 6X-FFF beams (Fig. 1). Boost irradiation (BI) was added to the original tumor bed and covered a 2.0-cm margin, with a daily dose of 2.65 Gy and up to a total dose of 10.6 Gy in four fractions, using 6–15 MeV electrons for all patients excepted those who had only pathologically ductal carcinoma *in situ* (DCIS) and for whom a surgical margin of more than 5 mm could be achieved. Organ at risks (OARs) including the heart, left anterior descending artery, lung and right breast were contoured in accordance with the European Society for Radiotherapy and Oncology guidelines [7].

Dose constraints were as follows: for left-side WBI, whole heart *V*_30_ < 5% and mean heart dose < 5 Gy; bilateral lung *V*_20_ < 5% and mean dose < 15 Gy; left anterior descending artery *D*mean < 10 Gy and *D*max < 20 Gy; right breast *D*_1cc_ < 10 Gy.

All treatment plans were designed and calculated by the Analytical Anisotropic Algorithm (AAA) of the Eclipse planning system (version 13.6, Varian Medical Systems, Palo Alto, CA). The calculation grid size was 2 mm.

### Deep inspiration breath-hold

Before irradiation, each patient was trained for DIBH by a radiation therapist. The radiation therapist instructed each patient to inhale and stop whenever possible with DIBH, and we used a real-time tumor-tracking radiotherapy system, SyncTraX (Shimadzu Co., Kyoto, Japan), to manage target motion because of respiration guided by one to three fiducial markers attached on the surface [8].

### Endpoint

The primary endpoint was the rate of occurrence of radiation dermatitis of Grade 3 or higher or pneumonitis of Grade 2 or higher within 6 months after the start of radiotherapy. The Common Terminology Criteria for Adverse Events version 4.0 (NCI CTCAE v4.0) were used to grade toxicity.

### Sample size

For a non-inferiority hypothesis, assuming a primary endpoint of 5% based on our historical patients who received 50 Gy in conventional fractions for breast cancer after breast-conserving surgery and a non-inferiority margin of 10%, a total sample of 25 patients was required, considering drop out of a few patients, to provide a statistical power of 80% and one-sided alpha of 0.05.

### Follow-up

During radiotherapy, a radiation oncologist saw the patients every week. Adverse events were evaluated at least every 1–2 months for the first 6 months and then at least every 3–4 months for 1 year. Chest X-rays and CT scans were performed if necessary.

## RESULTS

Twenty-five Japanese women were enrolled between February 2021 and November 2022 in this study. All of the enrolled patients completed the planned radiotherapy without interruption. The patients’ characteristics are shown in Table 1. The median age of the patients at study entry was 47 years. Follow-up for 6 months or longer was performed for all of the enrolled patient. So far, there has been no patient with locoregional recurrence or metastases.

**Table 1:** Patients’ characteristics

Age	Median (range)	47 (30–59) years
Performance status (ECOG)	0/1	*n* = 25/0
pStage (UICC 8th)	0/IA/IB/IIA/IIB	*n* = 2/16/0/6/1
Histology	IDC/DCIS/others	*n* = 21/2/2
Pathological tumor size	Median (range)	13.5 (2–31) mm
BI	Yes/no	*n* = 16/9

In only WBI, mean ± SD of whole heart *V*_30_ and mean heart dose, bilateral lung *V*_5_ and *V*_20_ and left anterior descending artery *D*mean and *D*max were 0 ± 0.2%, 0.9 ± 0.7 Gy, 15.8 ± 5.7%, 5.5 ± 2.3%, 3.6 ± 3.0 Gy and 8.3 ± 6.2 Gy, respectively.

Adverse events are shown in Table 2. The evaluation of adverse events showed that three patients (12.0%) had Grade 2 radiation dermatitis. No other Grade 2 adverse event was found and there was no patient with an adverse event of Grade 3 or higher. Two patients felt slight fatigue (Grade 1) but no patient had nausea during radiotherapy. No cardiac events have occurred to date. The non-inferiority margin was set as a 10% or less incidence of Grade 3 or higher radiation dermatitis or Grade 2 or higher pneumonitis within 6 months of starting radiotherapy, but both rates were 0%. Therefore, our hypothesis that the experimental treatment method is non-inferior compared with our historical results was confirmed.

**Table 2:** Number of patients with maximum adverse events according to CTCAE v4.0

**Adverse events**	**Maximum Grade *N* (%)**			
	G0	G1	G2	G3–5
Radiation dermatitis	0 (0.0)	22 (88.0)	3 (12.0)	0 (0.0)
Pneumonitis	23 (92.0)	2 (8.0)	0 (0.0)	0 (0.0)
Dry skin	18 (72.0)	7 (28.0)	0 (0.0)	0 (0.0)
Nausea	25 (100.0)	0 (0.0)	0 (0.0)	0 (0.0)
Fatigue	23 (92.0)	2 (8.0)	0 (0.0)	0 (0.0)

Of 16 patients who received BI, only one patient had Grade 2 radiation dermatitis. There was no significant difference in adverse events between patient with BI and patients without BI.

## DISCUSSION

The incidence of severe adverse events was lower than expected from past patients based on our experience of 3.2%, who were treated with conventional fractionated 3D-CRT with free breathing [9–13]. The results of this trial suggested that VMAT under the condition of DIBH in hypofractionated radiotherapy for left-sided breast cancer after breast-conserving surgery can be performed safely in Japanese women. To the best of our knowledge, this is the first report of a prospective clinical trial showing the safety of VMAT under the condition of DIBH in hypofractionated radiotherapy for WBI. The rates of severe adverse events in this trial were comparable to those in previous studies using more conventional dose-fractionation, sequencing schedules and techniques [11, 12, 14] (Table 3). The results of this trial were in agreement with the results of a previous study showing that HF-WBI caused milder skin reactions than did conventional fractionated WBI [10, 13]. Our results were also consistent with the results of a previous study showing that IMRT could reduce acute skin toxicity in conventional fractionated WBI compared with 3D-CRT [10]. There was concern about an increase in the incidence of radiation pneumonitis because of the increase in low-dose areas in the left lung, but there was no increase in the incidence of radiation pneumonitis in this trial.

**Table 3:** Summary of previous reports on postoperative radiotherapy for breast cancer

Authors	Total dose/fractions	Radiation method	Adverse events	
			Dermatitis Grade 3 or higher	Pneumonitis Grade 2 or higher
Nozaki *et al*. [11]	42.56 Gy/16 fractions for WBI ± 10.64 Gy/4 fractions for BI	3D-CRT under free breathing	0.00%	2.63%
Wang *et al*. [13]	50 Gy/25 fractions for WBI+ 10 Gy/5 fractions for BI	3D-CRT under free breathing	0.60%	3.00%
	43.5 Gy/15 fractions for WBI+ 8.7 Gy/3 fractions for BI	3D-CRT under free breathing	0.50%	2.20%
Chua *et al*. [14]	42.5/16 fractions or 50 Gy/25 fractions for WBI ± 16 Gy/8 fractions for BI	3D-CRT under free breathing	1.92%	0.20%
Nozaki *et al*. [12]	50 Gy/25 fractions for WBI ± 10 Gy/5 fractions for BI	3D-CRT under free breathing	n.a.	1.28%
Choi *et al*. [10]	50.4 Gy/28 fractions for WBI + 9 Gy/5 fractions for BI	3D-CRT under free breathing	n.a.	3.16%
	50.4 Gy/28 fractions for WBI, 57.4 Gy/28 fractions for BI	IMRT (SIB) under free breaths	n.a.	2.04%
Tang *et al*. [9] (our past study)	50 Gy/25 fractions for WBI+ 10 Gy/5 fractions for BI	3D-CRT under free breaths	1.10%	2.10%
The present study	42.4 Gy/16 fractions for WBI ± 10.6 Gy/4 fractions for BI	IMRT under DIBH	0%	0%

The reason for using VMAT under the condition of DIBH in patients with left-sided breast cancer is to reduce cardiac damage as much as possible. However, the results of our past planning study showed that the mean heart dose was lower than the 3D-CRT with free breathing and 3D-CRT under the condition of DIBH, but the reduction was 0.4–1.0 Gy, which was not very large [6]. Although Darby *et al*. [15] reported a 7.4% reduction in cardiac adverse events with a 1.0 Gy reduction in mean heart dose, cardiac adverse events are only evident after a long period. A comparative study would require a very large number of patients and a long observation period to show the superiority of this treatment to 3D-CRT under the condition of DIBH, and such a study seems unrealistic. Whether or not this treatment method should be applied to all patients with left-sided breast cancer should be considered in terms of cost-effectiveness. Radiation-induced heart injury is known to occur frequently in patients with risk factors [15], and it might therefore be better to use this treatment method only in patients with such risks.

In some cases, there may be no benefit from VMAT compared with 3D-CRT for OARs under the condition of DIBH. Therefore, the decision to use VMAT might need to be considered on a case-by-case basis.

As a limitation, because of the small number of cases and lack of long-term follow-up data in the present study, it has not been possible to determine whether radiation-induced cardiac damage is reduced or whether the local control rate is inferior.

## CONCLUSION

VMAT under the condition of DIBH in hypofractionated radiotherapy for left-sided breast cancer after breast-conserving surgery can be performed safely in a clinical setting.

## FUNDING

This work was supported partially by Health and Labour Sciences Research Grants (JPMH 23814582).

## CONFLICT OF INTEREST

All the authors declare that they have no potential conflicts of interest in this manuscript.
